# Ultrasonic evaluation of metoclopramide’s effect on gastric motility in emergency trauma patients

**DOI:** 10.3389/fphys.2023.999736

**Published:** 2023-05-10

**Authors:** Huan Lin, Jing-Jing He, Zhi-Shi Cai, Zhi-Wei Lu, Zhi-Jian Lin, Xian-Zhong Lin, Qiao-Wen Huang

**Affiliations:** ^1^ Department of Anesthesiology, Zhangzhou Affiliated Hospital of Fujian Medical University, Zhangzhou, China; ^2^ The Emergency Department, Zhangzhou Affiliated Hospital of Fujian Medical University, Zhangzhou, China; ^3^ Department of Anesthesiology, The First Affiliated Hospital of Fujian Medical University, Fuzhou, China

**Keywords:** gastric ultrasound, antral area, gastric emptying rate, gastric volume, metoclopramide

## Abstract

**Objective:** The present study aimed to use bedside ultrasound to evaluate the effects of metoclopramide on gastric motility in patients being treated for trauma in the emergency department.

**Methods:** Fifty patients underwent an ultrasound immediately after attending the emergency department of Zhang Zhou Hospital due to trauma. The patients were randomly divided into two groups: a metoclopramide group (group M, n = 25) and a normal saline group (group S, n = 25). The cross-sectional area (CSA) of the gastric antrum was measured at 0, 30, 60, 90, and 120 min (T = time). The gastric emptying rate (GER, $GER=-\left[\left({A_{area}}^{Tn}{{/A}_{area}}^{Tn-30}\right)-1\right]\times 100$ ), GER/min (GER divided by the corresponding interval time), gastric content properties, Perlas grade at different time points, T120 gastric volume (GV), and GV per unit of body weight (GV/W) were evaluated. The risk of vomiting, reflux/aspiration, and type of anesthetic treatment were also evaluated.

**Results:** The differences between the two groups in the CSA of the gastric antrum at each time point were statistically significant (*p* < 0.001). The CSAs of the gastric antrum in group M were lower than those in group S, and the greatest difference between the two groups occurred at T30 (*p* < 0.001). The differences between the two groups in GER and GER/min were also statistically significant (*p* < 0.001); those differences in group M were higher than those in group S, and the greatest differences between the two groups occurred at T30 (*p* < 0.001). There were no obvious change trends in the properties of the gastric contents and the Perlas grades in either group, and the differences between the two groups were not statistically significant (*p* = 0.97). The differences between the two groups in the GV and GV/W at T120 were statistically significant (*p* < 0.001), as was the risk of reflux and aspiration at T120 (*p* < 0.001).

**Conclusion:** When metoclopramide was used in satiated emergency trauma patients, it accelerated gastric emptying within 30 min and reduced the risk of accidental reflux. However, a normal gastric emptying level was not achieved, which can be attributed to the delaying effect of trauma on gastric emptying.

## 1 Introduction

For anesthesiologists, reflux and the aspiration of gastric contents have always been important focal points. Moos and Hansen (2008) stated that trauma is an important factor in aspiration pneumonia. Often, emergency trauma patients have residual gastric contents due to the ingestion of food before injury, the accidental swallowing of nasal and/or oral blood after injury, and delayed gastric emptying due to stress, pain, or the use of opioids (Murphy et al., 1997; Kao et al., 1998). During sedation or general anesthesia, such satiated patients are often at risk of aspiration due to a reduction in lower esophageal sphincter tension and the protective inhibition of the airway reflex (Vanner et al., 1992). Perioperative gastric ultrasound can be performed at a bedside ultrasound unit; it can safely, non-invasively, conveniently, and effectively evaluate the fullness of a patient’s stomach and the nature of their gastric contents (Van de Putte and Perlas, 2014; Van de Putte et al., 2017). It can also be used in the selection of an appropriate method for the anesthetic induction process and can reduce the risk of vomiting, aspiration, and related complications (Bouvet et al., 2011).

As a gastric motility-promoting drug, metoclopramide can accelerate gastric emptying. However, it remains controversial as to whether metoclopramide can be used to reduce the risk of pulmonary aspiration in patients with a high risk of reflux and aspiration. Clinically, it has not yet been used for the evaluation of emergency trauma patients. The present study, therefore, aimed to evaluate, using bedside ultrasound, the effects of metoclopramide on gastric motility within 120 min of emergency trauma surgery.

## 2 Methods

### 2.1 Study design

This study was a single-center prospective randomized double-blind parallel controlled trial. It was approved by the Ethics Committee of Zhang Zhou Hospital (no. 2019LW019) and registered with the China Clinical Trial Data Center (registration no. ChiCTR190022680).

### 2.2 Participants

Between November 2019 and January 2021, 50 patients undergoing emergency debridement in Zhang Zhou Hospital were enrolled, and written informed consent was obtained from the patients and their families.

Inclusion criteria were as follows: 1) healthy adults who planned to undergo emergency debridement; 2) 20–60 years of age; 3) 155–180 cm in height; 4) 45–75 kg in weight; 5) body mass index <30 kg/m2; 6) American Society of Anesthesiologists grade I–II; and 7) a cross-sectional area (CSA) of gastric antrum >5.5 cm^2^ (Van de Putte et al., 2017) measured at time T) = 0 (T0).

Exclusion criteria were as follows: 1) a history of diseases of important organs, such as diabetes, gastroparesis, liver and kidney dysfunction, and cardiopulmonary insufficiency; 2) contraindications for metoclopramide treatment; 3) taking opioids or drugs that affect gastric motility; 4) a history of esophageal, gastric, or upper abdominal surgery; 5) a need for immediate emergency surgery, resulting in insufficient study time (120 min); and 6) inability of the patient or their family to understand the study protocol.

### 2.3 Ultrasound procedure

After visiting the hospital’s emergency department, the patient was examined immediately by ultrasound (S-Nerve Series, Sono Co., Ltd., China, fitted with a 3–5 MHz convex array probe). The probe was placed in the sagittal position under the xiphoid process next to the upper abdomen, and the abdominal imaging mode was selected. The left lobe of the liver and the abdominal aorta served as anatomical marks on the section of the ultrasound image (Haskins et al., 2018). First, the patient was placed in a supine position and underwent a qualitative evaluation of the gastric antrum (to determine gastric contents, i.e., liquids and solids). Then, the CSA of the gastric antrum was measured (recorded as T0) during the interval of gastric peristalsis. The measurement range was from the subserosa to the serosa, and the average value of three measurements was taken as the result. The patient was then placed in a half-sitting/half-lying position (the head of the bed was raised by 45°), the aforementioned steps were repeated, and the inspection time was limited to 5 min. In accordance with the inclusion and exclusion criteria, the next stage of research was conducted for patients with a gastric antrum CSA of >5.5 cm^2^; otherwise, the trial was terminated. All patients who met the inclusion and exclusion criteria fasted (food and liquids) before the procedure and did not receive any opioids or medications that might affect gastrointestinal motility.

The ultrasound examinations were performed by three anesthesiologists with gastrointestinal ultrasound experience who had received special training. All measurement methods and procedures were carried out according to standardized methods (Bouvet et al., 2011).

### 2.4 Grouping

The sample size required for this study was estimated based on a trial of erythromycin in emergency trauma patients with a full stomach (Bouvet et al., 2016). In that trial, the gastric emptying rate (GER)/min of the emergency trauma patients was −0.05% ± 0.26% min^−1^, and the GER/min of erythromycin for the emergency trauma patients was 0.17% ± 0.30% min^−1^. Since both erythromycin and metoclopramide are gastric motility drugs, it was assumed that metoclopramide could produce the same gastric motility effect as erythromycin. The value of the reliability coefficient α was set to 0.05, and the power of the test was 0.8. Using PASS 11.0, it was calculated that 21 patients were required in each group. Based on the actual situation, 25 patients were enrolled in each group.

The patients were divided randomly into two groups: a metoclopramide group (group M, n = 25) and a normal saline group (group S, n = 25) using a random number table sealed in an opaque envelope. Metoclopramide and 0.9% sodium chloride were prepared and assigned to random numbers. The patients in group M were administered 10 mg of metoclopramide intravenously, while the patients in group S were given 1 ml of 0.9% sodium chloride intravenously.

### 2.5 Measurements

The aforementioned measurements were repeated at 30, 60, 90, and 120 min (T30, T60, T90, and T120, respectively). The antrum (Darwiche et al., 1999; Bouvet et al., 2011) and GER/min were expressed as the rate of gastric emptying in the corresponding period of time divided by the corresponding interval (Bouvet et al., 2016).

When the Perlas grade was 0 or 1 and the CSA of the gastric antrum was <3.4 cm^2^, it was defined as fasting (Cubillos et al., 2012; Perlas et al., 2016), and the risk of reflux and aspiration was considered to be low. When the Perlas grade was 2, regardless of the area of the gastric antrum, it was defined as a full stomach and the risk of reflux and aspiration was considered to be high. When the Perlas grade was 1 and the gastric antrum CSA was >3.4 cm^2^, it was defined as intermediate gastric content (Perlas et al., 2011). The formula for gastric volume (GV) in a semi-recumbent position was GV (ml) = [CSA(mm^2^) − 230]/4.6, where GV/W was the GV per unit of body weight. When GV/W > 1.5 ml/kg, it was considered a high risk for reflux and aspiration; when GV/W < 1.5 ml/kg, it was considered a low risk for reflux and aspiration.

### 2.6 Statistical analysis

Statistical analyses used SPSS 25.0, and GraphPad Prism 8 was used for mapping. The measurement data were expressed as mean ± standard deviation. The repeated-measurement data were compared between groups using repeated-measures analysis of variance, and independent measurement data were compared between two groups using an independent-sample t-test. Count data were expressed as the number of cases and compared between groups using a chi-squared test. The repeated-measures categorical data were compared using a generalized estimation equation. A value of *p* < 0.05 was considered statistically significant.

## 3 Results

The basic characteristics of the 50 patients included in this study were compared, and the differences were not statistically significant (Table 1) (*p* > 0.05).

**TABLE 1:** Demographic characteristics of patients in the two groups ( $\bar{x}$ ± SD and n = 50).

Item		Group M (n = 25)	Group S (n = 25)	Statistics	*p*-value
Age (years)		36.08 ± 8.29	33.24 ± 8.40	t = 1.20	0.24
Sex (male/female)		14/11	12/13	χ^2^ = 0.32	0.57
Height		163.48 ± 6.58	164.32 ± 6.94	t = −0.44	0.66
Weight		55.56 ± 8.03	54.36 ± 7.84	t = 0.54	0.60
BMI (kg/m2)		20.57 ± 2.01	20.01 ± 1.52	t = 1.13	0.27
ASA grade		10/15	11/14	χ^2^ = 0.08	0.77
Trauma site	Upper limb trauma (cases)	7	11	χ^2^ = 2.36	0.50
	Lower limb trauma (cases)	11	8		
	Extremity trauma (cases)	6	6		
	Other trauma (cases)	1	0		
Trauma score (ISS score) (point)		7.52 ± 1.81	7.88 ± 1.69	t = −0.73	0.47

**FIGURE 1:**
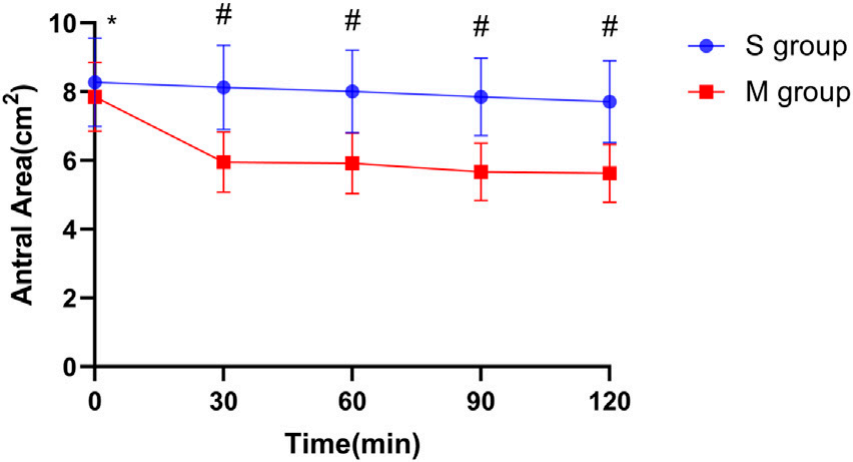
Changes in the cross-sectional area of the gastric antrum at different time points in two groups. Notes: The vertical line segment represents a range of “average value ± standard deviation”; * indicates that there was no significant difference between the two groups (*p* = 0.18); # indicates that the difference between the two groups was statistically significant (*p* < 0.001).

In group M, the CSA of the gastric antrum in the semi-recumbent position was smaller than that in group S, and the difference was statistically significant (*p* < 0.0001). The difference between the two groups was the greatest at T30 (*p* < 0.0001) (Figure 1).

**FIGURE 2:**
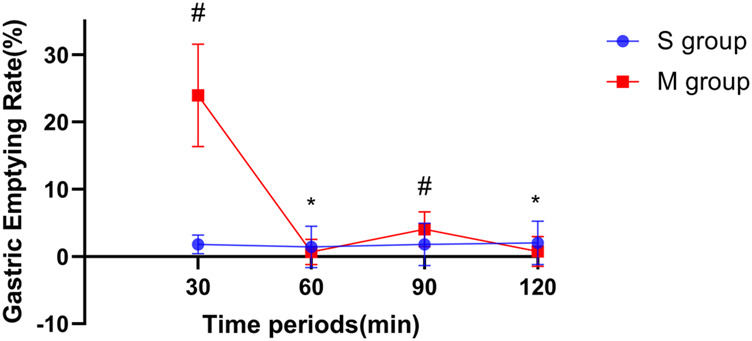
Comparison between two groups in the gastric emptying rate at each time point with an interval of 30 min. Notes: The vertical line segment represents a range of “average value ± standard deviation”; * indicates that there was no significant difference between the two groups (pT60 = 0.295 and pT120 = 0.112); # indicates that the difference between the two groups was statistically significant (*p* < 0.001).

The difference in GER every 30 min between the two groups was statistically significant (*p* < 0.0001); the GER was greater in group M than in group S, and the difference between the two groups was greatest at T30 (*p* < 0.0001) (Figure 2).

**FIGURE 3:**
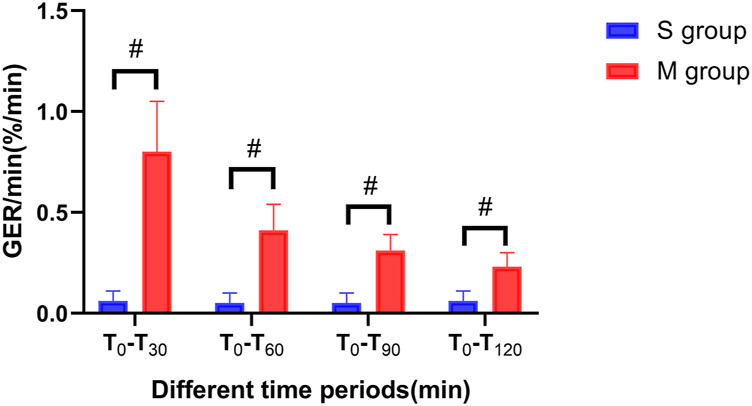
Comparison between two groups in the gastric emptying rate per minute at different time points. Notes: The vertical line segment represents a range of “average value ± standard deviation”; # indicates that the difference between the two groups was statistically significant (*p* < 0.001).

The difference in GER/min between the two groups was statistically significant (*p* < 0.0001); the GER/min was greater in group M than in group S, and the difference between T0 and T30 was the greatest at these time points (*p* < 0.0001) (Figure 3).

There were no significant differences between the two groups in terms of the properties of gastric contents (solids or liquids) and gastric emptying. The changing trend of the Perlas grade from grade 2 to grade 0 was not obvious, and the difference between the two groups was not statistically significant (*p* = 0.97).

The differences in GV and GV/W at T120 between the two groups were statistically significant (*p* < 0.0001) (Table 2).

**TABLE 2:** Comparison of GV and GV/W at T_120_ between the two groups ( $\bar{x}$ ± SD and n = 50).

Group	T_120_	
	GV (ml)	GV/W (ml/kg)
Group S	117.50 ± 25.90	2.17 ± 0.62
Group M	72.42 ± 18.29	1.38 ± 0.45
t	7.11	5.16
*p*	<0.001	<0.001

The difference between the two groups in the risk of reflux and aspiration at T120 was statistically significant (*p* < 0.0001) (Table 3). Two patients in group S had a bout of vomiting during the perioperative period, and no patients in group M vomited during the same period. This may have been related to the antiemetic effect of metoclopramide, but the difference was not statistically significant. A risk assessment was performed, and the corresponding anesthetic treatment was provided for the two patients in group S that had vomited. No reflux or aspiration occurred in either group.

**TABLE 3:** Comparison of the risk of T_120_ reflux and aspiration between the two groups (*n* = 50).

Group	T_120_	
	>1.5 mL/kg	<1.5 mL/kg
Group S	19	6
Group M	4	21
χ^2^	18.12	
*p*	<0.001	

## 4 Discussion

Darwiche et al. (1999) found that when healthy adults ate rice, the GER/min was 0.8% ± 0.25% min^−1^. Bouvet et al. (2016) evaluated the gastric emptying of patients with trauma before and after intervention and measured the GER before intervention at −0.05% ± 0.26% min^−1^. In the present study, the GER of the patients in group S at T120 was found to be 0.06% ± 0.05% min^−1^, which was similar to the results of Bouvet et al. but still significantly lower than that of healthy adults. Compared with group S, we measured the GER of patients in group M within T120 at 0.230.70% min^−1^, which was statistically significantly different. For T0–T30, T30–T60, T60–T90, and T90–T120, the GER results did not show any obvious changes in physiological digestion. The properties of gastric contents and the Perlas grades were not significantly different at T120.

A possible cause of this is the pharmacological properties of metoclopramide. Metoclopramide can accelerate gastric emptying by acting on dopamine receptors, and when it is used for intravenous injection, its onset time is just 1–3 min (Isola et al., 2020); as such, the gastric emptying effect was strongest at T0–T30. In the interval of T30–T60, the GER in group M was lower than that in group S. This was possibly caused by the serious effect of trauma on gastric emptying and the weakening of the gastric motility-promoting effect of metoclopramide. In the intervals of T60–T90 and T90–T120, the difference in GER was statistically significant, but compared with T0–T30, the GER decreased significantly. There were no significant differences in the properties of gastric contents and antrum scores in each period.

Trauma is an important factor in aspiration pneumonia. When GV/W > 1.5 ml/kg, the patient is considered to be at a high risk of reflux and aspiration. When GV/W < 0.8 ml/kg, the risk of reflux and aspiration is considered to be very low, and when GV/W is between 0.8 and 1.5 ml/kg, the risk of reflux and aspiration is considered to be low. In the present study, the GVc value in group S was 117.39 ± 25.94 ml, while GVc/W was 1.98 ± 0.57 ml/kg at T120. At the same time point (T120) in group M, GVd was 72.44 ± 12.24 ml, and GVd/W was 1.22 ± 0.31 ml/kg. The differences between the two groups were statistically significant, and the risk of reflux and aspiration was lower in group M. However, the CSA of the gastric antrum in both groups was greater than 3.4 cm^2^ at any time, which did not meet the fasting standard. Metoclopramide, as a commonly used antiemetic drug, can increase the tension of the lower esophageal sphincter without changing the compliance of the gastroesophageal junction (Mikami et al., 2016). Additionally, it helps reduce the reflux and aspiration of gastric contents and prevent aspiration pneumonia.

In terms of anesthetic management, among the 25 patients in group S, 19 had a high risk of reflux and aspiration, while 6 had a low risk; among the 25 patients in group M, 4 had a high risk of reflux and aspiration, and 21 had a low risk. For those patients with a high risk of reflux and aspiration, a protective anesthetic strategy was implemented to prevent reflux and aspiration during the perioperative period. For the low-risk patients, corresponding anesthetic treatments were given according to conventional methods. In the present study, a risk assessment was conducted, and the corresponding anesthetic treatment was implemented for these patients. No reflux or aspiration occurred in either of the two groups.

In terms of adverse reactions, metoclopramide was used for satiated emergency trauma patients at a dose of 10 mg, which was administered via a single intravenous injection. No serious adverse reactions occurred.

This study was a single-center study with a small sample size in which a single dose of a drug (10 mg) was used. There was no exploration of whether higher doses of metoclopramide alone or in combination with other gastric motility drugs were more effective in accelerating gastric emptying. The randomization method was used to ensure the average distribution of influencing factors (including some known and unknown influencing factors), but some unknown influencing factors were not included in the study. The primary focus of this study was the effect of metoclopramide on reducing the volume of gastric contents, but due to the limitations of the research protocol and sample size, the drug’s effect on reducing the incidence of aspiration pneumonia was not studied. It may be of greater clinical significance if a larger sample size study is used in conjunction with the reduction in the incidence of aspiration pneumonia as the main index.

## 5 Conclusion

When metoclopramide is used in satiated emergency trauma patients, it can accelerate gastric emptying and reduce the risk of accidental reflux within 30 min; however, it does not achieve normal gastric emptying levels, which may be related to delayed gastric emptying following trauma. Based on the antiemetic effect of metoclopramide, these findings can provide a basis for the preoperative treatment of such patients.

## Data Availability

The original contributions presented in the study are included in the article/Supplementary Material; further inquiries can be directed to the corresponding author.
